# Heart transplantation in single-ventricle physiology with failed fontan circulation

**DOI:** 10.1186/1749-8090-8-S1-O136

**Published:** 2013-09-11

**Authors:** E Delmo Walter, R Hetzer

**Affiliations:** 1 Cardiothoracic Surgery, Deutsches Herzzentrum Berlin, Berlin, Germany

## Background

Failed Fontan circulation who underwent heart transplantation, with focus on indications, timing, strategies, risk factors and long-term outcome.

## Methods

Between 1986 and 2011, 179 children underwent heart transplantation for cardiomyopathy and end-stage congenital heart disease. Fifteen patients had single-ventricle physiology and underwent Fontan procedure. Five patients (mean age 6.6±1.2, range 2-14, years old) had failed Fontan circulation. Interval between Fontan procedure and failing circulation was 8-14 years. Indications for heart transplantation were ventricular failure (n=4), and protein-losing enteropathy (n=1).

## Results

The technical strategy during transplantation included pulmonary artery reconstruction, redirection of the venous drainage and oversizing of the donor heart. The patient with early Fontan failure is alive 20 years post-transplantation. One patient with a late Fontan failure had acute graft dysfunction, increased pulmonary vascular resistance, chronic organ congestion and perioperative hemorrhage. A biventricular assist device was implanted but the patient died 24 hours later. Two patients with mild pulmonary hypertension died 1 month and 10 months, respectively, after heart transplantation and biventricular assist device implantation to support the failing graft. The last patient with a late Fontan failure is alive and well 8 years after transplantation. Early mortality is 20% while late mortality is 40%. At a mean follow-up of 18 years, overall survival is 40%. A risk factor for mortality is increased pulmonary vascular resistance.

## Conclusion

Heart transplantation in failed Fontan circulation, though technically challenging, may be offered in this difficult group of patients who are otherwise hopeless.

